# Advances and challenges in regenerative therapies for abdominal aortic aneurysm

**DOI:** 10.3389/fcvm.2024.1369785

**Published:** 2024-06-04

**Authors:** Calvin L. Chao, Brandon Applewhite, Nidhi K. Reddy, Natalia Matiuto, Caitlyn Dang, Bin Jiang

**Affiliations:** ^1^ Division of Vascular Surgery, Department of Surgery, Northwestern University Feinberg School of Medicine, Chicago, IL, United States; ^2^ Department of Biomedical Engineering, Northwestern University McCormick School of Engineering, Chicago, IL, United States

**Keywords:** abdominal aortic aneurysm, regenerative medicine, vascular surgery, cellular therapy, biomaterials

## Abstract

Abdominal aortic aneurysm (AAA) is a significant source of mortality worldwide and carries a mortality of greater than 80% after rupture. Despite extensive efforts to develop pharmacological treatments, there is currently no effective agent to prevent aneurysm growth and rupture. Current treatment paradigms only rely on the identification and surveillance of small aneurysms, prior to ultimate open surgical or endovascular repair. Recently, regenerative therapies have emerged as promising avenues to address the degenerative changes observed in AAA. This review briefly outlines current clinical management principles, characteristics, and pharmaceutical targets of AAA. Subsequently, a thorough discussion of regenerative approaches is provided. These include cellular approaches (vascular smooth muscle cells, endothelial cells, and mesenchymal stem cells) as well as the delivery of therapeutic molecules, gene therapies, and regenerative biomaterials. Lastly, additional barriers and considerations for clinical translation are provided. In conclusion, regenerative approaches hold significant promise for *in situ* reversal of tissue damages in AAA, necessitating sustained research and innovation to achieve successful and translatable therapies in a new era in AAA management.

## 1 Introduction

Abdominal aortic aneurysm (AAA), defined as a localized dilation of the infrarenal aorta to greater than 3.0 cm, is a progressive degenerative disease of the aorta that culminates in aortic rupture, massive hemorrhage, and subsequent death. Globally, nearly 170,000 deaths per year can be attributed to AAA (1). Despite this devastating toll on human health, there are still no pharmacologic therapies to treat AAA (2, 3). Screening studies have suggested the modern prevalence of AAA is 1%–3% and potentially greater in high-risk populations (4, 5). Irrespective of sex, incidence of AAA increases with advanced age (6, 7). This is a critical consideration in an aging global population, as longer patient survival affords prolonged AAA expansion. While some recent reports suggest a decreasing prevalence of AAA, the mortality after ruptured AAA remains elevated at nearly 80% (4).

Considering the dearth in pharmacological approaches to treat AAA, the current gold standard for AAA management is either open aortic surgery to replace the diseased infrarenal aorta with a synthetic (PTFE, Dacron) graft or endovascular aneurysm repair (EVAR), which has become the preferred treatment modality, to achieve sac exclusion via precise intraluminal deployment of a covered stent-graft (8). Despite remarkable advancements in surgical technique, particularly in the endovascular space, no non-surgical therapy has successfully slowed or halted AAA growth. While EVAR offers some perioperative advantage, both modalities are still associated with significant procedural risk, cost, and long-term sequelae including reintervention (9). Thus, strategies to improve long term survival in AAA patients remain lacking. Efforts should be directed to treat AAA lesions not yet large enough to qualify for surgical repair and prevent the need for surgery altogether. Regenerative approaches hold promise to treat AAA directly rather than simply removing the diseased tissue from circulation.

**Figure 1:**
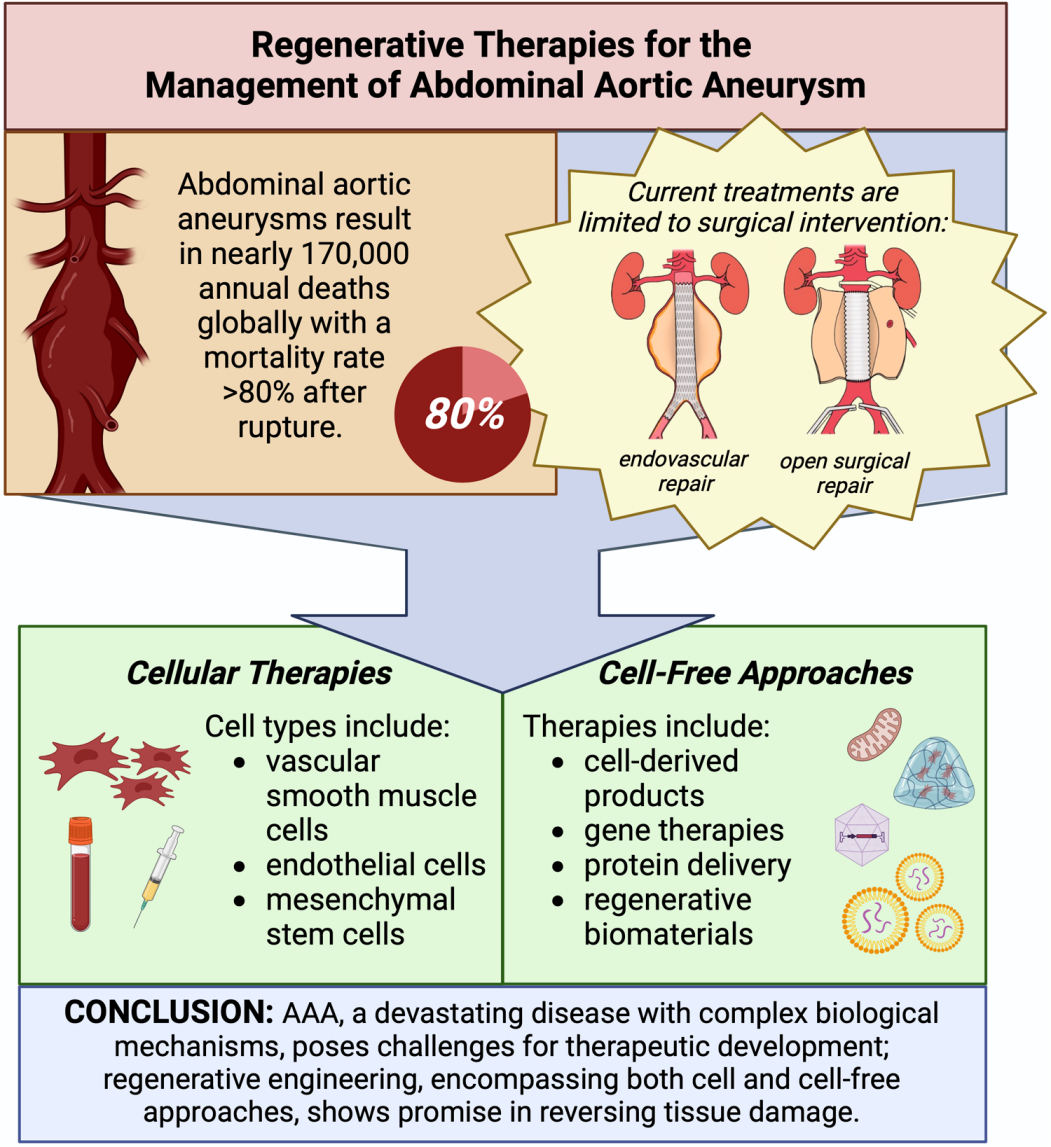
Regenerative therapies for the management of abdominal aortic aneurysm. Illustrations in top right panel adapted with permission from [Kyriacou et al. (15)], licensed under CC BY 4.0, https://doi.org/10.1177/1750458920947352. Created with BioRender.com.

Regenerative engineering is an emerging discipline that combines tenets from cell biology, materials science, and biomedical engineering to develop treatments to repair, replace, and regenerate terminally damaged tissues that cannot be healed via classical methods, making it a promising approach to combat aortic degeneration. While pharmacological therapies may slow AAA progression, regenerative engineering offers the possibility of healing the aneurysmal tissue by encouraging the formation of new tissue. This can be accomplished *in situ* by cell delivery, biomaterial implantation, and local delivery of exogenous proteins such as growth factors and antibodies or by combining one or more of these to achieve a synergistic effect (10, 11). For example, cells can be localized by culturing them on a scaffold or within a hydrogel prior to implantation (12). Besides providing a substrate for cell attachment, biomaterials also provide biomechanical input to influence cell fate and phenotype (13). Similarly, biomaterials enable the controlled delivery of exogenous proteins, protecting those proteins from rapid degradation and providing sustained release to directly target the damaged tissue (14). The current paradigm for regenerative engineering in AAA management is using cell delivery to blunt the progression of aneurysms. In the following sections, we provide a synopsis of the various regenerative engineering approaches which have been proposed for AAA treatment including cell therapy, cell-derived products, controlled protein delivery, gene therapy, and regenerative biomaterials critiquing the practical and technical aspects of each method (Figure 1). We will conclude by describing the challenges which have impeded clinical translation of these experimental therapies and suggesting new frontiers to which regenerative strategies might be incorporated into AAA management.

## 2 Current understanding of AAA pathogenesis and therapeutic development

Previous research endeavors have attempted to elucidate the intricate mechanisms that govern the progression of AAA growth to identify suitable targets for non-invasive therapies. This has been the cornerstone of pharmacologic strategies with the hope of identifying a single putative driver of aneurysmal pathology. However, to date no pharmaceutical intervention has proven successful in the clinical realm, most likely due to a still incomplete mechanistic understanding of AAA pathogenesis and its differential genesis including genetic predisposition, atherosclerosis, and inflammatory etiologies. Conversely, a primary benefit of a regenerative strategy is the ability to repair existing damage rather than address the root cause. Nevertheless, a comprehensive understanding of the underlying molecular and cellular mechanisms seen in AAA pathogenesis and ensuing histopathologic changes is crucial in the design of effective regenerative therapies. Studies examining AAA pathogenesis through various means have collectively unveiled numerous factors that have been extensively reviewed by Golledge et al. (3). These encompass a spectrum of elements, including but not limited to the following: hemodynamics and mechanical stress, endothelial injury, extracellular matrix (ECM) remodeling, inflammation and immune responses, vascular smooth muscle dysfunction, and genetic mechanisms. While many of the regenerative approaches highlighted later in this review are purportedly etiology-agnostic by addressing these elements, there likely remain strategies that are more suitable for one phenotype vs. another. For instance, AAA secondary to hereditary connective tissue orders may require direct addressment of underlying gene polymorphisms with varying severities (16). Similarly, mycotic aneurysms are a particularly distinct phenotype that mandate addressment of an underlying latent infection. A continued limitation remains the varying, but all somewhat limited, pre-clinical models of AAA that only partially recapitulate all features of human disease (17). Ultimately, though an idealistic regenerative therapy may strive for a lofty one-size-fits-all approach, integrating etiology and phenotype into therapy design likely holds the greatest promise for clinical translation.

### 2.1 Characteristics of AAA and potential targets for regeneration

**Figure 2:**
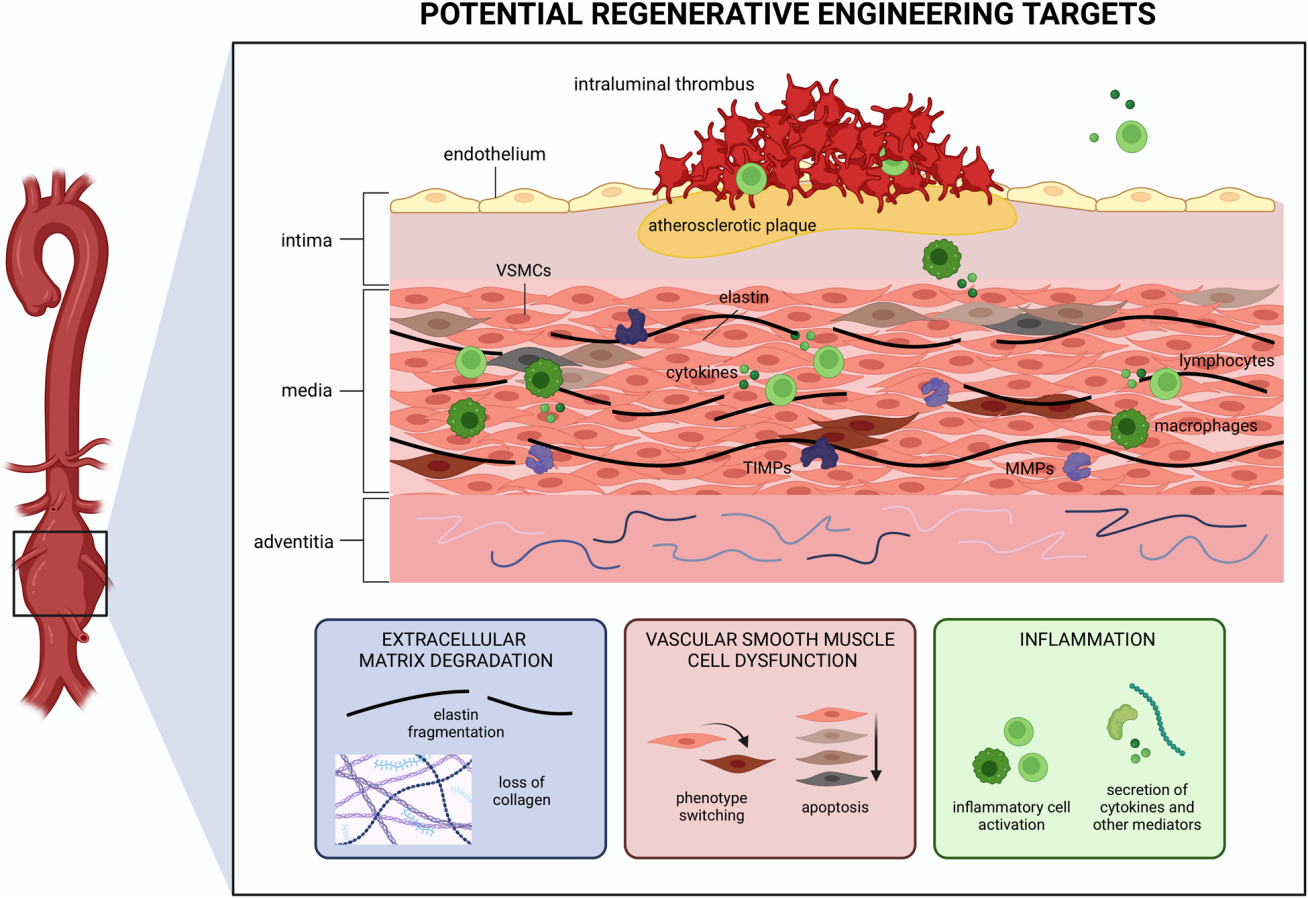
Schema of cellular and molecular processes involved in abdominal aortic aneurysm (AAA) pathogenesis. Extracellular matrix (ECM) degradation, vascular smooth muscle cell (VSMC) dysfunction, and inflammation are present throughout the aortic wall. Each presents a potential target for regenerative medicine strategies. Created with BioRender.com.

The hallmark histopathological features of AAA are: (1) degradation of the ECM, especially of the elastin fibers; (2) apoptosis and subsequent loss of vascular smooth muscle cells (VSMCs), which are responsible for providing vascular tone and contractility; and (3) accumulation and activation of inflammatory cells such as macrophages, which in aggregate, lead to degeneration of the vascular wall and aneurysm formation (18, 19). Additionally, AAA also often present with a multilayered intraluminal thrombus (ILT) (18). Each of these features represents a target for therapeutic intervention using regenerative strategies (Figure 2). Addressing any of these facets may successfully improve other features in parallel as AAA formation is an intricate multifactorial process. In this section, we describe how regenerative engineering may be applied to regenerate AAA tissue and function by targeting one or more of these features.

The vascular ECM plays a significant role in vascular development and homeostasis (20). It is mainly comprised of collagen and elastin fibers, both of which impart the fundamental biomechanical behavior required for vascular function (21). AAA tissue is marked by proteolytic fragmentation of elastin fibers which jeopardizes the integrity of vascular wall. Two primary protease families—serine proteases and activated matrix metalloproteinases (MMPs)—are significantly associated with AAA progression (19). The main MMPs involved in AAA pathogenesis are the gelatinases MMP2 and MMP9 (22). In healthy tissues, these enzymes are responsible for maintaining ECM homeostatic turnover by working in concert with tissue inhibitors of metalloproteinases (TIMPs). When this delicate balance shifts to an increase in MMPs or reduction in TIMPs, tissue catabolism occurs. Furthermore, ECM destruction has been shown to provoke VSMC death (18). Therefore, regenerative strategies that target ECM degeneration might reverse the course of AAA formation, and approaches that encourage ECM synthesis, deposition, and crosslinking are all plausible ways to regenerate the AAA wall. This includes transplantation of secretory cells to produce new ECM and delivery of growth factors such as transforming growth factor-beta (TGF-β) that promote ECM production. Alternatively, biomaterials can also be used to induce a localized foreign body response to inspire ECM accumulation at the aneurysm lesion (23). Lysyl oxidase is a key enzyme responsible for the posttranslational crosslinking of collagen and elastin, allowing the aortic wall to resist dilatation and rupture (24, 25). Promoting ECM crosslinking has thus been recommended as a strategy to combat AAA development using interesting approaches such as lysyl oxidase overexpression or *in situ* ECM crosslinking (24–26).

VSMCs are considered the functional unit of the vasculature given their role in maintaining vascular tone, facilitating vessel contraction, and regulating ECM synthesis (3). VSMC hypocellularity is considered a hallmark of AAA. Although it is unclear whether VSMC dysfunction is a result or a cause of AAA formation, VSMC differentiation into pathological phenotypes with altered contractile properties and secretory profiles further exacerbate aortic wall weakening and aneurysm expansion (3, 19). Additionally, VSMC senescence is increasingly recognized as a contributor to AAA pathogenesis (27, 28). Regardless, regenerative engineering can be employed to repopulate the vascular wall either directly by cell transplantation or indirectly by methods that prevent VSMC apoptosis and transdifferentiation, induce VSMC proliferation, or encourage resident stem and progenitor cells to differentiate into VSMCs.

It is postulated that this unregulated inflammation leads to the destruction of the vascular wall. Profound immune cell infiltration including neutrophils, macrophages, mast cells, natural killer cells, dendritic cells, B cells, and T cells is a hallmark of aneurysmal tissue. Specifically, polymorphonuclear leukocyte activation and death result in the release of granule contents, including proteases, oxidant peptides, myeloperoxidase, and pro-inflammatory mediators such as IL-8 (19). Human studies highlight distinct phenotypes of monocytes and macrophages in AAA development (19). Circulating monocytes of patients with AAA exhibit higher expression levels of lymphocyte function-associated antigen1 (LFA1) and CD11b compared to healthy individuals (19). Macrophages accumulate predominantly in the adventitia and ILT, contributing significantly to the inflammatory environment (19). In addition to immune cell infiltration, adventitial tertiary lymphoid organs (TLOs), which are organized lymphocytic neo-granulomas with a germinal center composed of B cells, have also been identified in tissue (18). Regenerative engineering approaches to combat inflammation are therefore an intuitive approach to restore AAA tissue. While dampening inflammation may be viewed as a way to slow AAA progression rather than restore tissue, inflammation can be harnessed to promote tissue regeneration. For example, macrophage polarization toward the pro-healing M2 phenotype is a common approach in regenerative engineering (29). Adoptive transfer of CD4 + lymphocytes was shown to have therapeutic effects on vascular remodeling in arteriovenous fistula models (30). Regenerative therapies can and should be optimized to manage AAA by modulating the immune response.

### 2.2 Pharmaceutical strategies to attenuate AAA

Promising preclinical studies have led to several clinical trials and observational studies seeking to attenuate AAA growth. Presently, all completed placebo-controlled drug trials have resulted in negative findings (31). These discouraging results are potentially explained by limitations in trial design, including small sample size and insufficient duration of follow-up. This lack of translation may also be due to incomplete understanding of AAA pathogenesis, underscoring the need for novel targets and therapies, including regenerative medicine approaches. Nevertheless, contemporary randomized controlled trials offer valuable insights into the challenges associated with developing new therapeutics for the clinical management of AAA.

The initial selection of antibiotic therapies was driven by their effects on inhibiting MMP activity involved in AAA pathogenesis (32). To date, 3 trials have investigated doxycycline as a potential therapy, all with no impact on AAA growth or need for repair (33–35). Additionally, prior investigations into AAA pathogenesis had identified *Chlamydophila pneumoniae* as a potential driver of AAA expansion (36). As such, macrolide antibiotics garnered significant interest to target *Chlamydophila pneumoniae* (37). However, 1 trial investigating azithromycin has also found no significant impact on AAA expansion and furthermore no correlation between *Chlamydophila pneumoniae* antigen and disease burden (38). Additionally, 2 small trials investigating roxithromycin found potential reduction in AAA expansion (39, 40). However, both trials were limited by modest patient enrollment and a subsequent meta-analysis by Golledge et al. found antibiotic therapies had limited efficacy in limiting AAA growth or reducing rates of AAA repair or rupture (41).

Initial antihypertensive strategies to attenuate AAA expansion focused on propranolol given early reports that beta blockade may reduce AAA rupture in pre-clinical models as well as retrospective studies of human patients (42). To date, two trials have investigated propranolol without positive results and were additionally marred by significant rates of dropout due to intolerance of beta blockade (42, 43). Driven by pre-clinical animal models and observational population studies suggesting angiotensin converting enzyme (ACE) blockade reduced AAA, ACE inhibition was the next pharmacologic strategy to undergo clinical trials (31, 44). A single trial investigating perindopril found superior reduction in blood pressure in the ACE inhibitor arm but no significant difference in aneurysm growth rate (45). Most recently, angiotensin II receptor blockers (ARB) have also undergone evaluation as a potential pharmacologic strategy. Again, a single trial investigating telmisartan found no difference in AAA growth but again increased rates of hypotensive symptoms (46). These negative findings were further substantiated by the above meta-analysis by Golledge et al. (41). Together, these findings suggest that hemodynamic factors alone do not adequately protect against AAA expansion or rupture.

Numerous trials have demonstrated the benefit of statin therapy on all-cause mortality and cardiovascular morbidity and mortality (47). However, no large randomized controlled trial has demonstrated similar efficacy in the attenuation of AAA. Nevertheless, the pleiotropic effects of statin therapy are worth mention given robust cohort studies. Pooled analyses suggest statin therapy not only reduces growth rate but also reduces rupture risk and improves survival after elective repair (48). Several mechanisms have arisen as contributors to these observed benefits including modulation of endoplasmic reticulum stress and oxidative stress, inhibition of MMPs and maintenance of ECM components, as well as anti-inflammatory effects (49). Given the numerous cardiovascular comorbidities endemic to the AAA patient population, statin therapy is likely to remain a mainstay in mitigating cardiovascular risk. Further exploration of statin-specific mechanisms and identification of therapeutic targets for AAA is warranted.

Several other strategies to attenuate AAA expansion have reached clinical trials, each focused on different aspects of AAA biology. Despite the well described inflammatory changes in the aneurysmal aortic wall, only one clinical trial has utilized an anti-inflammatory strategy. A single trial investigating varying doses of pemirolast, a mast cell inhibitor, revealed no differences in AAA growth at 1 year (50). Pre-clinical models of AAA have indicated anti-lipid therapies may potentially attenuate AAA expansion as well as inflammatory infiltration (51). Fenofibrate, a member of the fibrate class, was subsequently selected for evaluation and remains the only lipid-modifying agent to undergo a randomized clinical trial to date; no difference was observed in AAA growth at study conclusion (52). The role of aortic thrombus has been identified as a contributor to AAA expansion, potentially driven by both cellular signaling and matrix remodeling, as well as changes to mechanical forces (53, 54). The lone trial evaluating anti-thrombotic therapies utilized the antiplatelet agent ticagrelor and found no significant difference in AAA diameter at 1 year (55). Notably, ticagrelor had no significant impact on intraluminal thrombus volume and the role of intraluminal thrombus remains to be fully elucidated.

Given the numerous negative trials to date, as detailed above, new strategies to attenuate AAA expansion and reduce AAA rupture are critically needed. As in the case of prior pharmacologic trials, a regenerative approach may retain a focus on simply slowing the underlying destructive processes and thus delay or obviate the ultimate requirement for surgical intervention. However, the advantage of a regenerative strategy is also the potential to fully reverse the degenerative features of aneurysmal disease and serve as the sole therapy for this pathology. In the remainder of this review, we highlight promising and emerging regenerative therapies for AAA.

## 3 Cell-based regenerative therapies for AAA

**Figure 3:**
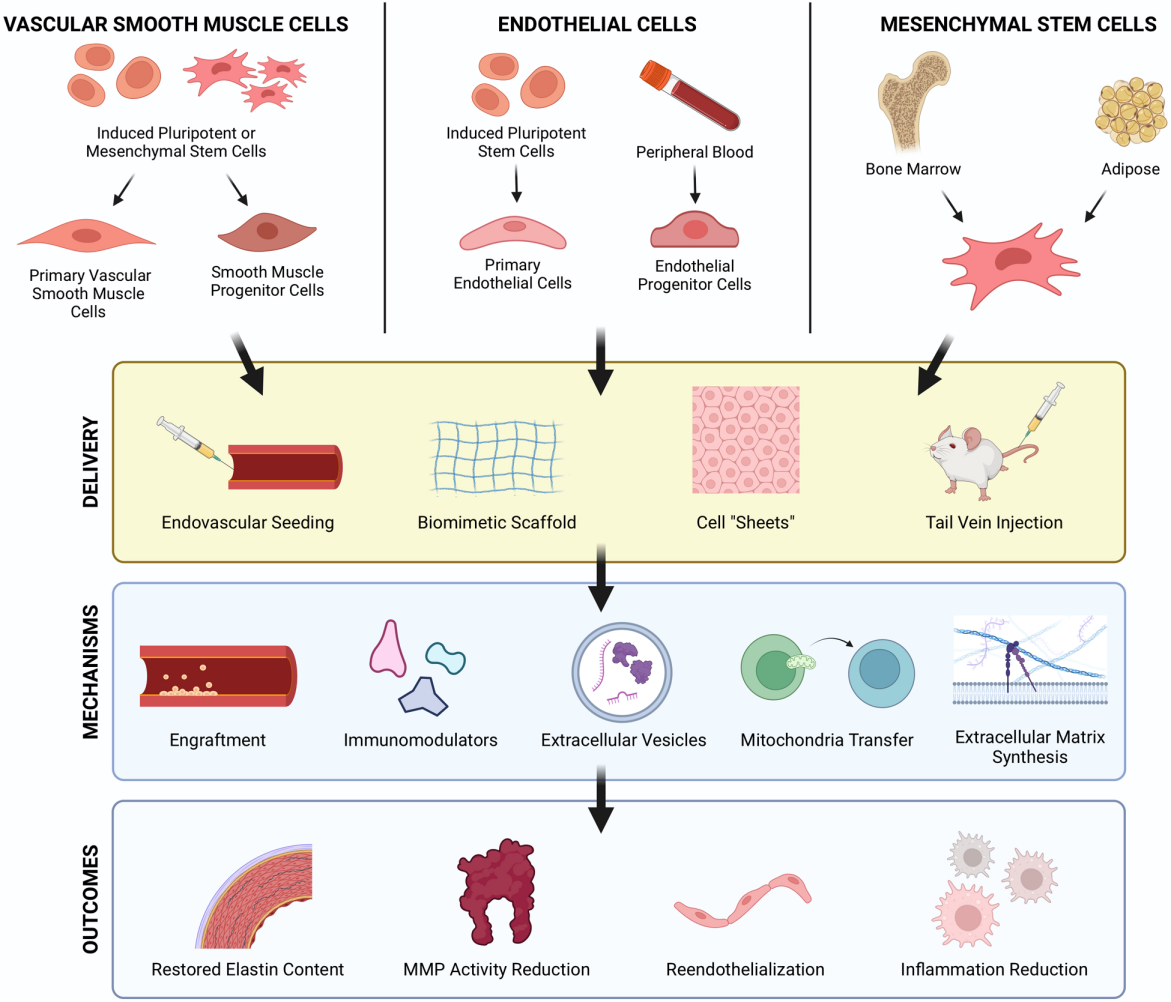
Cellular therapy strategies to regenerate vascular tissues in AAA. Cell therapies using VSMCs, ECs, and MSCs have each demonstrated therapeutic benefits for AAA treatment. These cells can be derived from iPSCs or collected from peripheral blood and allogenic/autologous tissue sources such as bone marrow and adipose tissue. Route of delivery, intravascular and periadventitial, are both feasible approaches with their own respective advantages and challenges. Cells exert regenerative effects via several mechanisms including direct engraftment, immunomodulation, paracrine signaling, and ECM synthesis which result in restored elastin, reduced protease activity, reendothelialization, and reduced inflammation. Created with BioRender.com.

Cell transplantation has been promoted as a regenerative therapy for damaged tissues and organs for decades. Originally, it was posited that exogenous, healthy cells could engraft in tissues where they could replace void space and encourage regeneration. Studies now show that delivered cells largely elicit their benefits through paracrine effects. Cell therapy for AAA treatment was first reported over two decades ago when Allaire et al. overexpressed TIMP-1 in syngeneic rat VSMCs prior to transplantation (56). Since then, an assortment of cells from distinct lineages have been used to treat AAA including VSMCs, endothelial cells (ECs), and stem and progenitor cells, each with their purported benefits for AAA repair (Figure 3).

### 3.1 Vascular smooth muscle cells or progenitors

The apoptosis of medial VSMCs and the subsequent decrease in VSMC density in the aorta is a hallmark of AAA, making VSMC transplantation an intuitive strategy to regenerate aneurysmal aortas. Allaire et al. first described VSMC delivery to determine whether the presence of VSMCs prevents AAA formation in a rat xenograft model (57). Endoluminal seeding of VSMCs at the time of xenograft implantation prevented aneurysm growth and elastinolysis which could be attributed to a shift in the proteolytic balance toward inhibition. In a follow-up study, cells were seeded 2 weeks after xenograft implantation to allow for aneurysm formation (58). VSMC infusion preserved aortic diameter and elastin content by reducing proteolysis and macrophage infiltration while increasing fibrous collagen transcripts. However, the mechanism of action of VSMCs in this model is unclear as the cells were introduced within the lumen and risked potential loss upon perfusion. Surely VSMCs were observed in the intima/ILT after 1 week, suggesting a paracrine mechanism for the observed improvements and indicating the ILT as a biological target. Nearly a decade later, Park et al. tested what they termed “vascular smooth muscle cell-like progenitor cells” (VSMC-PCs) as a therapy for AAA in a rat elastase model (59). These cells were obtained from muscle MSCs that were differentiated *in vitro* with PDGF-bb before transplantation. While this report did not provide any information regarding the effect of cell therapy on the aneurysm morphology, there was a reduction in proteases.

Most recently, Mulorz et al. compared the therapeutic effects of periadventitial delivery of induced pluripotent stem cell (iPSC) derived smooth muscle progenitors (iPSC-SMPs) and primary human aortic SMCs in a murine elastase model using a commercially-available collagen sponge (Vitene™) covered with a polycaprolactone (PCL) film in a seminal translational study (60). Collagen sponges are biomimetic scaffolds that exhibit features of the native ECM including a fibrillar morphology, porosity, and integrin binding sites for cell attachment which may be beneficial for cell delivery. Interestingly, primary SMC treatment significantly reduced aortic diameter compared to iPSC-SMPs and was superior in maintaining freedom from AAA despite iPSC-SMPs persisting longer in the aneurysm wall. This study illustrates the delicate balance researchers must deliberate when deciding which differentiation state to use for their cell therapy as progenitor cells may have more longevity while adult cells may possess a more therapeutic secretome. It also demonstrates the feasibility of using a periadventitial scaffold to deliver cells to aneurysms.

In aggregate, these studies provide a framework for VSMC delivery for AAA treatment. While the mechanism is still unclear, they demonstrate the therapeutic benefits of VSMCs in preventing AAA progression by reducing proteolysis. Future studies will be needed for VSMC delivery to determine the optimal number, differentiation state, route, and timing of cell transplantation.

### 3.2 Endothelial cells

Reendothelialization and induction of angiogenesis via cell transplantation of ECs and endothelial progenitor cells (EPCs) has been proposed as a tissue engineering strategy to treat vascular disease (61–64). EPCs are a subset of circulating hematopoietic cells that possess the unique potential to differentiate into ECs and contribute to neoangiogenesis. Interestingly, both increased and decreased numbers of EPCs have been reported in patients with AAA (65). Transplantation of ECs and EPCs has been effective in treating arteriovenous fistula stenosis (66), vascular injury (67, 68), and hindlimb ischemia (62). These therapeutic benefits derive from their paracrine action rather than assembly into new vasculature as they secrete molecules which regulate vascular function and promote angiogenesis such as nitric oxide, vascular endothelial growth factor (VEGF), basic fibroblast growth factor (bFGF), and heparin (67–70). Recent evidence has identified a role of hypoxic signaling and reduced vasa vasorum density as a key factor in AAA progression (71, 72). In a recent case study, a patient was noted to have an entire avascular region of AAA tissue upon gross examination which lacked vasa vasorum and presented severe disruption of the elastic fibers, histologically (73). As such, EC delivery is an intuitive treatment paradigm for AAA.

There has only been one report on EC delivery for AAA to date. Franck et al. measured the impact of endovascular EC delivery on AAA prevention and stabilization in a rat xenograft model (74). ECs were either seeded at the time of xenograft implantation (prevention) or 14 days after (stabilization). EC delivery before and after AAA formation was effective in reducing diameter increase, demonstrating their potential as regenerative therapy to prevent further AAA dilation. Further analysis showed that EC therapy led to the establishment of a functional endothelium and a robust vascular wall with preserved elastin and collagen matrix. To determine the contribution of exogenous ECs to the observed neointimal regeneration, the authors conducted an elaborate experiment showing that ECs recruit tissue resident cells to facilitate remodeling, ruling out circulating EPCs as contributors to the newly formed intima. These findings reinforce the idea that the therapeutic benefits of cell delivery arise from paracrine effects rather than tissue engraftment. Noting the translational barriers of using adult ECs as a cell therapy, the authors conducted a similar study using “outgrowth endothelial cells” (OECs), a form of EPCs, and obtained similar results. While this work shows the promise of ECs as a cell therapy for AAA treatment and signifies the importance of a functional endothelium in AAA repair, future studies are still needed to explore alternative delivery routes, especially considering the difficulty of seeding cells endovascularly in the presence of an ILT. Surely, periadventitial EC delivery has been shown to be effective despite its distance from the endothelium (75). Additionally, periadventitial delivery may be advantageous in regenerating the vasa vasorum thereby reducing medial hypoxia and preventing further degeneration. Alternatively, another avenue are strategies to direct endogenous ECs and EPCs to regenerate aneurysmal tissue *in situ* rather than transplanting cells.

### 3.3 Mesenchymal stem cells

Mesenchymal stem cells (MSCs) are adult multipotent stem cells that reside in different tissues throughout the body including bone marrow, muscle, adipose tissue, placenta, and umbilical cord and maintain the potential to differentiate into osteogenic, chondrogenic, adipogenic, and neuronal lineages. Compared to embryonic stem cells, MSCs are safer as they reduce the risk of teratoma formation and pose less ethical concerns regarding sourcing (76). MSCs have thus been at the forefront of regenerative cell therapies due to their widespread availability, differentiation potential, and inherent healing properties as undifferentiated cells. MSCs have shown curative effects for a plethora of inflammatory diseases and as cellular agents in vascular regenerative medicine (77–81). Their immunomodulatory effects and differentiation potential have positioned MSCs as frontrunners in regenerative therapies for AAA, with the majority of reports describing the effects of MSC transplantation in slowing aneurysm progression. In the following sections, we will critique MSC therapies that have been applied to experimental AAA models stratified by the tissue source, bone marrow or adipose tissue, and provide insight into the efficacy and ultimate translatability of these approaches.

#### 3.3.1 Bone marrow derived MSCs

Bone marrow derived MSCs (Bm-MSCs) account for ∼0.01% of bone marrow mononuclear cells. As such, Bm-MSCs must be expanded *in vitro* to obtain adequate cell numbers for regenerative therapies (82). Still the properties of Bm-MSCs such as ease of isolation, rapid expansion, amenability to cryopreservation, and immune-privilege for allogeneic therapies make them worthy for clinical translation. Countless clinical trials have demonstrated the safety of Bm-MSCs in other applications (81). Bm-MSC therapy as a treatment for AAA has only recently reached the clinical realm (83); however, there have been a number of experimental and preclinical studies that indicate their promise as a potential AAA treatment.

Hashizume et al. first reported the use of Bm-MSC therapy for AAA in an angiotensin II (AngII) model using ApoE^−/−^ mice (84). Allogenic Bm-MSCs were first isolated from femurs and expanded *in vitro* using temperature-responsive well plates which enabled the cells to be transplanted as continuous sheets that could be applied directly to the adventitial surface. This method is advantageous as it preserves cell-cell and cell-ECM contacts which are essential to cell viability and optimal function and enables periadventitial delivery without laborious biomaterial fabrication. Transplantation of Bm-MSC sheets reduced the diameter of the infrarenal aorta and inflammation. Cell tracking located BM-MSCs in the aorta 28 days after transplantation, which raises the question of whether engraftment is a true phenomenon. Turnbull et al. also reported engraftment of Bm-MSCs after 1 week following injection and endovascular seeding in a porcine model (85). However, the biological insights of this study are limited as it did not include a control group in which an aneurysm was created without cell treatment. Schneider et al. evaluated the efficacy of Bm-MSC therapy in a rat xenograft model (86). Endovascular seeding of Bm-MSCs into the lumen of xenograft aneurysms 2 weeks after surgery significantly blunted the diameter increase and enhanced ECM content compared to vehicle controls. Interestingly, there was a dramatic reduction in monocyte/macrophage infiltration, highlighting the immunomodulatory effects of Bm-MSCs. Cell tracing revealed that delivered Bm-MSCs survived for a week after delivery but were not observable at 1 month. The transplanted cells were localized to the ILT, indicating it as an obstacle for endovascular delivery to the wall but also supporting the ILT as a viable target. Using a dose-relationship experiment, the authors showed that a single dose of one million Bm-MSCs was sufficient to stabilize the aneurysm whereas multiple doses of VSMCs were necessary to achieve the same effect, indicating that MSCs possess enhanced regenerative potential compared to adult cells.

**Figure 4:**
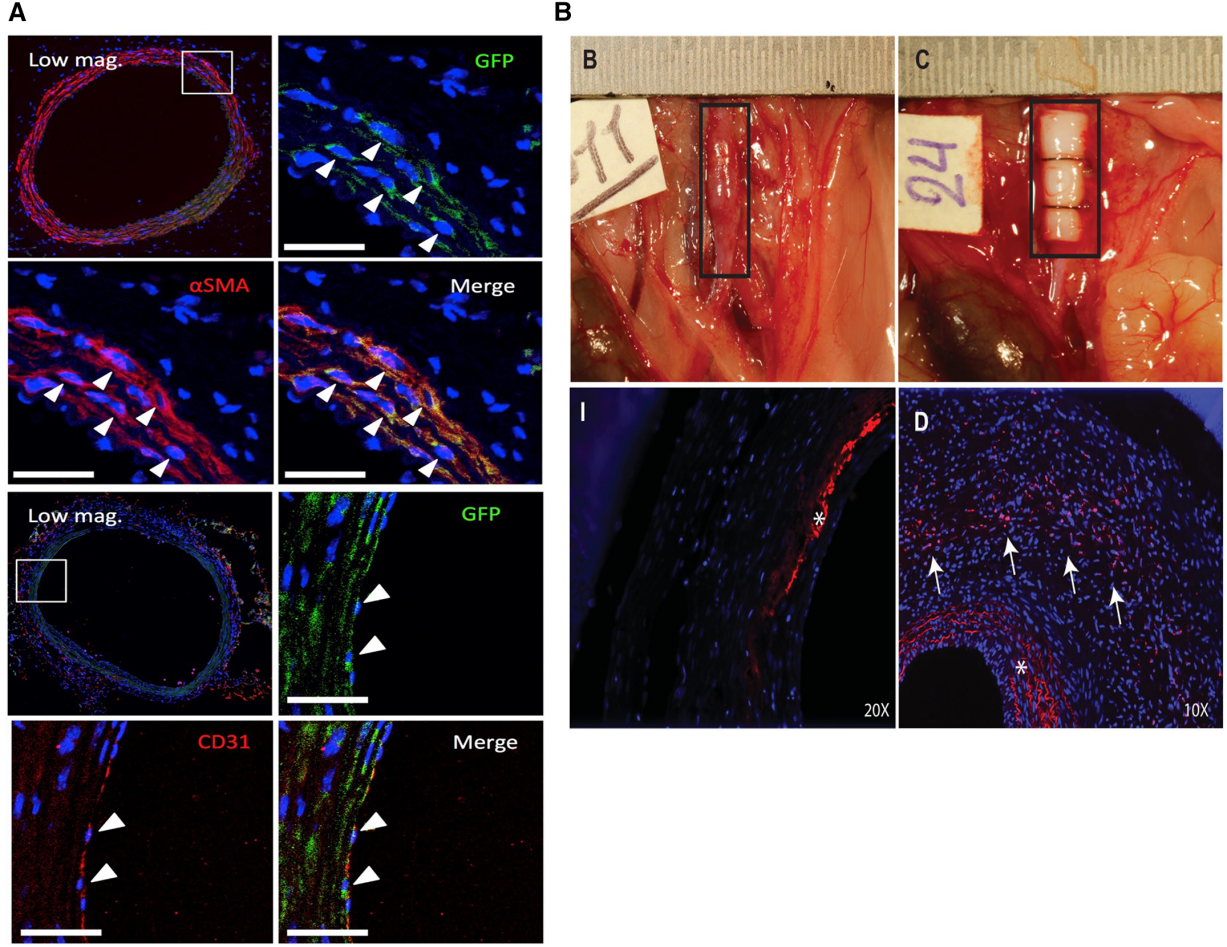
Fate of MSCs after periadventitial delivery. (**A**) Engraftment and differentiation of MUSE (GFP+) cells into SMCs (αSMA+, top) and endothelial cells (CD31+, bottom) 3 weeks after periadventitial delivery. Reprinted from (87) with permission from Elsevier, © 2018 by The American Association for Thoracic Surgery, https://doi.org/10.1016/j.jtcvs.2018.01.098. (**B**) Periadventitial delivery of ADSCs using recombinant collagen peptide patches (top right). Labeled ADSCs (red, arrows) could be visualized throughout the aortic wall after 14 days (bottom right) but not bare control patches (bottom left) (* = autofluorescence of the elastic laminae). Reprinted from (88) with permission from Elsevier, © 2018 Wiley Periodicals, Inc., https://doi.org/10.1002/jbm.a.36445.

Hosoyama et al. utilized a specialized subset of MSCs known as multilineage-differentiating stress enduring (MUSE) cells which express the embryonic surface marker stage specific embryonic antigen (SSEA)-3 in an elastase/CaCl_2_ model in severe immunocompromised mice (87). Despite their embryonic origin, these cells are purported to be non-tumorigenic and have rapid doubling time which makes them ideal for clinical translation. Multiple injections of MUSE cells most effectively prevented aneurysm growth and increased medial elastin compared to non-MUSE (SSEA-3^−^) cells and normal Bm-MSCs. A single injection of MUSE cells at day 0 was also superior to the other groups. Interestingly, MUSE cells were shown to colocalize with both SMA and CD31, showing their multipotency and capability for direct regeneration (Figure 4A). MUSE cells were shown to home to the aneurysm site from the adventitial side, suggesting they transport through the vasa vasorum. These findings emphasize the importance of cell sorting when designing future Bm-MSC therapies for AAA as Bm-MSCs contain a heterogenous population of cells with differential therapeutic properties. They also provide support for intravenous injection as a feasible delivery method for MSCs due to their ability to home to injury sites.

#### 3.3.2 Adipose derived MSCs

Adipose tissue is another reliable source for MSCs. Compared to Bm-MSCs, Adipose-derived MSCs (Ad-MSCs) can be isolated in higher abundance with less morbidity in a minimally-invasive procedure. Like Bm-MSCs, Ad-MSCs are profoundly immunomodulatory and anti-inflammatory and have been subject to numerous clinical trials (89). Despite still lacking clinical approval, several studies have demonstrated the promise of Ad-MSCs as a regenerative therapy for AAA over the last 10 years.

Tian et al. evaluated the effects of Ad-MSC therapy on AAA in a rat CaCl_2_ model after performing a series of *in vitro* and *ex vivo* experiments, showing that they improve elastin content and reduce MMP activity (90). In this study, cells were introduced to the aorta via the carotid artery. In contrast, recognizing the ILT as an impediment for intravascular delivery, Blose et al. utilized a periadventitial approach to deliver Ad-MSCs in an elastase model by connecting a sponge to the infusion catheter, effectively reducing aneurysm progression and preserving the elastic lamellae (91). Xie et al. delivered human Ad-MSCs via the tail vein in a mouse elastase model, beginning therapy at the time of elastase treatment (92). Intravenous Ad-MSC therapy had a profound anti-inflammatory effect reducing macrophage infiltration, polarizing macrophages toward the M2 phenotype, and increasing the proportion of regulatory T cells. These reductions in inflammation were associated with blunted aneurysm dilation over 2 weeks compared to controls. Transplanted cells were observed solely in the lung 1 day after injection and were no longer present after 4 days, suggesting that Ad-MSCs exhibit a systemic anti-inflammatory effect.

Employing a classical tissue engineering approach, Parvizi et al. developed perivascular scaffolds from a recombinant collagen peptide to deliver rat Ad-MSCs to the adventitia in a rat elastase/CaCl_2_ model (88). The recombinant collagen peptide consists of a repeat of human type 1 collagen along with an RGD sequence, thereby recapitulating features of the vascular ECM and facilitating cell adhesion, respectively. Ad-MSCs were seeded on the scaffolds for one day prior to transplantation and were transplanted at the time of elastase treatment. The scaffolds were fully intact after 2 weeks *in vivo* and labeled Ad-MSCs were shown to migrate from the adventitia into the media (Figure 4B). Only seeded scaffolds prevented dilation while there was a significant reduction in elastin and SMA expression and increase in macrophage infiltration with the scaffold alone while there was no difference in the Ad-MSC scaffold group compared to the sham. In all, this study highlights the utility in using engineered scaffolds to enhance the effects of Ad-MSC as a translatable local therapy, however a direct comparison between cells alone and cells seeded within scaffolds would be insightful.

## 4 Cell-free regenerative approaches

**Figure 5:**
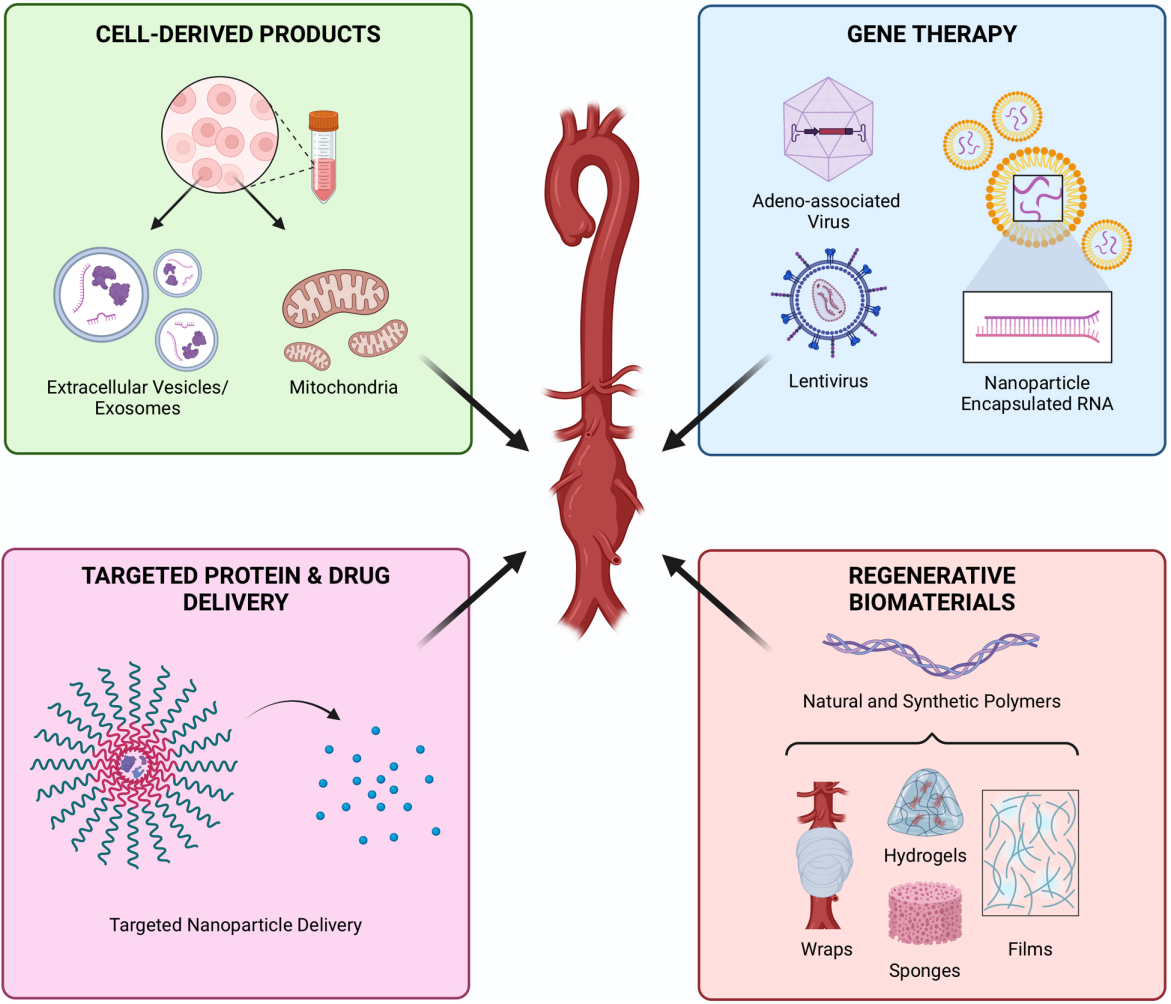
Cell-free regenerative approaches to attenuate AAA. Products derived from cells, including extracellular vesicles or mitochondria, offer the therapeutic paracrine effects of cells while avoiding some of the translational barriers of cell therapies. Gene therapy offers precise control of molecular mechanisms. Controlled delivery of proteins and drugs provides targeted and sustained therapeutic effects, while protecting payloads from rapid degradation in the inflammatory environment. Biomaterials are typically paired with a molecular or cellular payload but can also offer regenerative potential alone. Created with BioRender.com.

While cell therapy is well suited for the biology of AAA, cell-free approaches offer an alternative path to AAA regeneration, circumventing the barriers to translation that often hinder cell therapies. Cell-derived products, gene therapies, controlled protein delivery, and regenerative biomaterials are all options to promote AAA repair without the use of living cells (Figure 5). Instead, these approaches elicit therapeutic responses in the resident cells to accomplish regeneration. Compared to cell therapies, these approaches are often more specific, focusing on a particular target to accomplish healing and offering more precise control of the healing process. In the following section, we will review the alternative regenerative approaches to cell therapy that have been prescribed for or may be well suited to AAA treatment, offering critiques on previous studies and providing recommendations for the design of these treatments.

### 4.1 Cell-derived extracellular vesicles

Instead of live cell transplantation, cells can be used as *in vitro* factories to produce cell-derived therapeutics such as extracellular vesicles (EVs) (93–95). Exosomes, a subset of EVs, transport intracellular cargo outside the cell thereby playing an important role in intercellular communication (96). Specifically, exosomes carry an assortment of proteins and miRNA, which can promote AAA regeneration (97, 98). MSCs are often used as the cell sources for production of EVs. Chen et al. isolated EVs from MSCs to treat AngII-infused AAA mouse models thereby inhibiting AAA formation and improving survival (99). While MSC-EV-treatment helped inhibit neutrophil extracelluar trap (NET) mediated ferroptosis in SMCs *in vivo*, MSC-EVs were unable to inhibit NET-induced ferroptosis *in vitro.* Thus, the findings suggested that EV-treatment may not directly inhibit ferroptosis but help prevent NET formation and AAA development, though the exact mechanism requires further investigation. In addition to Bm-MSCs, Hu et al. isolated exosomes secreted by Ad-MSCs (ADSC-exos) and evaluated their effects in Ang II AAA (100). ADSC-exos were shown to reduce elastin degradation via exosomal miR-17-5p. Spinosa et al. demonstrated that EV treatment showed a decrease of inflammation and cytokine levels equivalent to MSC treatment (101). Further investigation of the specific mechanism of EV action demonstrated that this protective effect occurs through miR-147-mediated downregulation of IL-17 and HMGB-1. Surface modification of EVs can increase their tissue specificity and improve therapeutic effect. Sajeesh et al. modified EVs with cathepsin K (CatK) binding peptides (CKBP), noting CatK is highly expressed on the surface of ECs and SMCs in AAA (102). Modification with CKBP increased uptake and elicited a pro-regenerative effect on cytokine-activated SMCs isolated from an elastase-induced AAA rat model, however no *in vivo* study was performed.

In summary, EV-based therapies propose a new potential avenue for AAA regeneration through inflammation downregulation, reduction of elastin degradation, and inhibition of SMC activation. Such therapy might also be superior to cell-based therapies through the opportunity for targeted tissue-specific modification and low immunogenicity. However, the mechanism of action of such therapies and further investigation of specific cargoes that EVs carry are essential for further development of EV-based therapies for AAA regeneration.

### 4.2 Gene therapy

Another nascent field, gene therapy, has the potential to significantly improve clinical outcomes for AAA therapy. By using the power of gene manipulation such as overexpression or gene silencing through miRNA, siRNA, and viral vectors, gene therapy offers a novel approach to encourage AAA regeneration. Yan et al. studied the effect of the amphipathic protein-mediated nanoparticle delivery of siRNA targeting NF-κB (103). Hyaluronic acid (HA)-coated p5RHH nanoparticles were used to knock down either the p50 or p65 subunit of NF-κB in an elastase-induced model and a TGF-β blockade model. Interestingly, the delivery of nanoparticle-encapsulated siRNA targeting the p65 subunit was only effective at the early stage of post-elastase perfusion, while the p50 subunit silencing was beneficial even at 5 days post-perfusion. Overall, the silencing of different NF-κB subunits helped reduce AAA progression and rupture.

Zhao et al. demonstrated that knockdown of miR-33-5p led to increased expression of ABCA1 and activation of PI3K/Akt pathway, while transfection with siRNA targeting ABCA1 decreased phosphorylation of PI3K and Akt (104). Although the relation between the PI3K/Akt pathway and AAA is unclear, the authors hypothesized it modulates AAA formation by regulating cholesterol efflux. Tao et al. studied the cellular senescence of VSMC in human AAA specimens, revealing AngII induces VSMC senescence by downregulation of Sirt1 (105). The authors also demonstrated that miR-199a-5p mediates Sirt1 expression and thus identified it as a potential target for AAA gene therapies, along with miR-34, miR-455-3p, and miR-125b-5p which are also upregulated in AAA patients. Nevertheless, the role of such miRNAs and their molecular mechanisms require further investigation before they may be used for AAA regenerative gene therapies.

In addition to siRNAs, viral vectors open another avenue for gene therapy. Adeno-associated virus (AAV) is commonly used in gene therapy due to its efficiency in gene delivery, low risk of triggering an immune response, and long-term expression of a therapeutic gene (106). Li et al. presented evidence for lysyl hydroxylase 1 (LH1) being a potential gene target for AAA as human specimens and Ang II mice experience reduced LH1 expression, leading to the activation of proinflammatory process, increased MMP activity, and VSMC apoptosis (25). AAV-LH1 treated mice exhibited decreased AAA formation and rupture. Even though the findings presented evidence for AAA alleviation, gene therapy alone was not sufficient to completely regenerate dissecting AAA, suggesting gene therapies may need to be combined with other interventions to increase therapeutic efficacy. Zhao et al. demonstrated that BAF60c (a subunit of the SWItch/sucrose nonfermentable chromatin remodeling complex (SWI/SNF) is reduced in human AAA samples and murine Ang II and elastase AAA models and upregulation preserved healthy VSMC phenotype and suppressed VSMC inflammation (107). Overexpression of BAF60c in mice using an AAV did not improve the survival rate but significantly reduced the maximum aortic diameters and AAA development.

Long noncoding RNAs (lncRNA) represent an additional gene product with potential as a therapeutic target. Encouraging pre-clinical studies have utilized knockdown or knockout of lncRNAs with great effect. Zhang et al. previously identified upregulation of the lncRNA plasmacytoma variant translocation 1 (PVT1) in human AAA tissue and AngII induced murine AAA; lentiviral knockdown of PVT1 in an AngII murine model attenuated aortic expansion and suppressed VSMC apoptosis, matrix degradation, and inflammatory cytokine profile (108). Similarly, Li et al. identified the lncRNA H19 as one the most upregulated transcripts in the AngII and elastase murine models of AAA, a Yucatan mini-pig aneurysm model, as well as end-stage human disease (109). Subsequent knockdown in AngII and elastase murine models reduced AAA growth rate with reduced VSMC apoptosis mediated by interactions with hypoxia-induced factor 1α. This role of lncRNAs on VSMC behavior may hold particular promise in AAA biology. Ahmed et al. identified the lncRNA nuclear paraspeckle assembly transcript 1 (NEAT1) as a critical regulator of VSMC phenotype; *in vivo* knockout of NEAT1 significantly reduced VSMC proliferative phenotype, albeit in a non-aneurysmal model (110). These lncRNAs are merely a fraction of a rapidly growing database of lncRNAs and we anticipate continued identification of transcripts suitable for therapeutic targeting in AAA (111).

The unique setting of the vascular system presents some limitations for the use of novel and efficient gene editing systems like Clustered Regulatory Interspaced Short Palindromic Repeats (CRISPR) and the CRISPR-associated Proteins (Cas) proteins. The need for targeted therapy and delivery to vascular cells is challenged by the constant blood flow and changing environment of the vasculature. Another concern is off-target effects that are often difficult to predict. Nevertheless, a recent study done by Zhang et al. presented a lipid-based and hydroxyl-rich gene vector system with high transfection efficiency and stability (112). They developed CHO-PGEA/ pCas9-sgFbn1 nanoparticles targeting Fibrillin-1 and analyzed their efficiency *in vitro* and *in vivo*. *In vivo* studies demonstrated that AngII infusion significantly increased the uptake of nanoparticles by aortic tissues. Moreover, the delivery system did not demonstrate any toxic effects on organs, thus revealing a new potential avenue for gene therapy of vascular diseases.

In summary, gene therapy approaches are a promising route for AAA regeneration. However, current reports are typically mechanistic, investigating the role of a certain gene and pathway in AAA formation and progression, rather than focusing on clinical translation of these therapies. While such studies greatly contribute to our understanding of AAA formation and development mechanisms and identify novel targets for AAA regeneration, more translational studies will be useful to advance the field of regenerative therapies for AAA. In the future, more efforts should be directed to developing local delivery methods to enhance the therapeutic relevance of gene therapy approaches.

### 4.3 Targeted protein and drug delivery

Soluble signals are a main component of the tissue engineering triumvirate. Protein and growth factor delivery have been proposed to direct cell fate and ultimately exact tissue regeneration (14, 61, 113). However, growth factor therapeutics do not come without their own risks. Besides potential for immunogenicity, dosing and pharmacokinetics must be precisely controlled to reduce off-target effects and avoid tumor formation. Exogenous protein delivery is also hampered by their inherent lability and instability, as they are rapidly denatured in the physiological environment, losing their activity. To combat this, proteins are often combined with a biomaterial vehicle to provide controlled delivery, to preserve their activity and localize their effects (11, 114–116). Bai et al. evaluated the regenerative impact of an injectable hyaluronic acid/sodium alginate hydrogel loaded with TGF-β in a murine CaCl_2_ and rat vein patch model (117). TGF-β promotes AAA regeneration by inducing ECM production and dampening inflammation (118–121). This treatment effectively improved wall thickness and elastin integrity in the mouse model which was associated with increased transforming growth factor activated kinase (TAK1) and pSMAD2 positivity, indicating a TGF-β dependent mechanism. The hydrogel remained visible in histology at day 14, but there were no signs of cell infiltration and tissue integration. Also, the feasibility of intramural injection in a human aneurysm is unlikely due to the potential for rupture. Still, this study presents an interesting alternative approach which paves a new direction for regenerative therapies based on growth factor and cytokine signaling. More soluble factors can be screened for AAA regeneration as well as develop biomaterial delivery methods to control their presentation.

Although systemic pharmacological treatments have not been proven effective in the clinical management of AAA, local delivery of these agents using biomaterials can improve tissue regeneration by reducing the activity of proteins detrimental to tissue repair in the pathological microenvironment. In the context of regenerative engineering, these drugs are combined with a biomaterial delivery vehicle to improve the pharmacokinetics, and subsequently their safety and efficacy. For instance, as evidenced by multiple clinical investigations, doxycycline garnered significant interest as a synthetic MMP inhibitor. However, systemic administration is associated with off-target effects. To overcome this, Yamawaki-Ogata et al. developed electrospun nanofibers which released doxycycline slowly over 2 months without any toxic effects to measure the effects of local MMP inhibition in an angiotensin mouse model (122). Nosoudi et al. described the use of anti-elastin nanoparticles loaded with batimastat, a hydroamate-based MMP inhibitor, to determine its feasibility as an intravascular therapy that would traffic to the aneurysm site by targeting elastin in a rat CaCl_2_ model (123). Nanoparticles are advantageous as they protect their payloads from degradation in the physiologic environment and confer sustained release behavior; however, these particles emptied their loads by 1 week and, thus, had to be delivered weekly. These particles were shown to accumulate in the aorta while immunoglobulin-tethered controls did not. Long term, they improved histological features and prevented aortic dilation compared to nanoparticles without drug. Overall, these reports suggest local MMP inhibition is an effective strategy for AAA repair and regeneration by preventing ECM degradation and reducing inflammation. Future studies can combine local delivery of MMP-inhibitors with other regenerative strategies to improve AAA regeneration.

Controlled endovascular delivery technologies have demonstrated promise in large animal models and are perhaps the most advanced along the clinical translation pathway. Simionescu et al. delivered 1–3, 4, 6-pentagalloylglucose (PGG) via a weeping endovascular balloon to stabilize elastin and collagen in a swine AAA model (elastase/collagenase and CaCl_2_) with not only attenuation of AAA but also reduction in aortic diameter (124). This has since been delivered to human patients without obvious toxicity, though impact on aneurysm growth remains to be seen (125).

### 4.4 Regenerative biomaterials

Biomaterials are often combined with cells or other therapeutics before implantation; however, biomaterials alone often possess their own therapeutic effect due their inherent bioactivity and can be used to direct the behavior of endogenous cells. While this is especially true for natural biomaterials which contain domains which promote cell attachment and integrin signaling, such as ECM-derived polymers (i.e., collagen, gelatin, decellularized tissues), synthetic polymers can also be functionalized to control cell behavior (23). Natural and synthetic polymers are often combined to exploit the properties of each. These polymers can be fabricated into a slew of formats including sponges, foams, hydrogels, fibers, and films, all of which are amenable for periadventitial interventions that can be easily applied to the aneurysm lesion. Unlike current surgical options, these materials degrade over time, leaving by newly formed tissue, making them exciting alternatives to regenerating AAA.

The primary features of AAA pathogenesis are thinning of the vascular wall, persistent inflammation, and VSMC loss. Appropriate biomaterial selection would encourage wall ECM production and VSMC proliferation while attenuating inflammation to regenerate the vascular wall. Polyethylene glycol (PEG) hydrogels have attracted increasing interest due to their tunability and amenability to functionalization (126–128). PEG hydrogels are “blank slates” and can be functionalized with peptides and growth factors and designed with protease-sensitive crosslinks which enables cell-mediated degradation. Protease sensitivity might be an attractive feature in the context of AAA in which MMP activity is upregulated. PEG is also non-immunogenic, which is pertinent to AAA as any additional inflammation might worsen the disease state. In contrast, natural polymers are readily degraded by cells and do not require any complex chemistries but can still be modified if desired. While these polymers are often immunogenic, the foreign body response may be advantageous in AAA as it promotes local ECM production. Citrate-based biomaterials possess potent antioxidant properties which might be advantageous in combating AAA inflammation (129). Another interesting biologic-free approach is to design these biomaterials to sequester endogenous proteins and cells instead of delivering them which would be helpful in clinical translation (130). To date, there are no reports of using a biomaterial alone as a therapy for AAA regeneration besides vehicle controls in cell studies, but acellular biomaterials might be more effective, cheaper, and easier to translate.

## 5 Perspectives for clinical translation

Despite countless innovations and experimental therapies to promote AAA repair, few regenerative approaches have reached clinical trials and none have received final clinical approval (83). Before advancing to the clinic, it is imperative to demonstrate the safety, efficacy, and mechanism of action of these treatments in animal models. As such, clinical translation has been limited by variability among preclinical experimental studies, especially among animal models and treatment administration paradigms. Studies utilizing clinically-relevant large animal models have also been scant. Other challenges include the practical and technical considerations that accompany regenerative therapies such as scalable manufacturing. Lastly, there is a need for consistent collaboration among scientists, engineers, and physicians to determine a suitable treatment approach (i.e., administration route and timing) that aligns with clinical practice along with continued experimentation to unravel the underlying biology of AAA pathogenesis. In the following sections, we will elaborate on these obstacles to translation and prescribe some recommendations for future work to ultimately see a regenerative therapy clinically-approved for AAA.

### 5.1 Preclinical evaluation

Regardless of the approach, the ultimate success of any regenerative therapy for AAA relies on a standardized route to translation. Variability among experimental models, species, and study designs make it difficult to compare the efficacy of new treatments. Animal models of AAA remain imperfect, and no single model captures both the anatomic specificity and all histopathologic changes observed in human AAA. Models can broadly be categorized as dissecting or non-dissecting aneurysms. Dissecting models, such as the popular angiotensin II model, rely on generation of intimal tears and can importantly recapitulate aneurysmal rupture and subsequent death but often manifest outside of the infrarenal segment and may better reflect residual dissection after type B aortic dissection in human patients (17). Resultantly, this remains a significant limitation as the etiology and clinical management of post-dissection aneurysms is certainly distinct from degenerative AAA. Alternatively, non-dissecting models, such as the elastase or CaCl_2_ models, can induce progressive transmural dilation with anatomic specificity but lack the important clinical endpoint of rupture (17). Additional considerations include presence of an ILT, concomitant atherosclerosis, and inflammatory infiltration. The previously described rat xenograft model is another non-dissecting model that promotes both ILT and early immune cell infiltration with a similar limited capacity for rupture (131). The implantation of a decellularized guinea pig aorta reliably generates AAA and the associated void of VSMCs has made this model an attractive testbed for cellular therapies (74). Models also require some ongoing trigger for progression of pathology with consequent stabilization or healing after cessation, though some groups have demonstrated continuous progression of AAA by prolonging the initial trigger or new stimulus (132, 133). Each model should be considered complementary in fully capturing the human disease state and recognizing their limitations is paramount in evaluating any regenerative therapy.

Current approaches evaluate whether a potential therapy can stop aneurysm formation or slow progression rather than regenerating damaged tissue. The timing of intervention will govern the indication of any individual regenerative engineering approach. As such, it is necessary to develop strategies that align with the current clinical paradigm. Delivery of any therapy for AAA must integrate the existing framework for patient identification, repair strategies, and surveillance. While certain therapies may be more suited for early intervention, more drastic approaches may be needed once the aneurysm has dilated past a certain threshold. Similarly, assessment of regenerative therapies must be guided by clinically relevant endpoints as cellular or molecular markers alone may be insufficient given the limited translational success thus far. Aortic remodeling after EVAR offers some insight to favorable endpoints. Though EVAR is not inherently a regenerative therapy, the diseased aorta remains in place, and sac regression alone may predict long-term success (134, 135). Continued integration of advanced imaging modalities and other clinical parameters will remain imperative in assessing both surgical and regenerative therapies.

### 5.2 Source of regenerative materials

Cell therapies using cells of different phenotypes and lineages have shown promise as therapeutics in aneurysm treatment in numerous preclinical studies (Table 1) but have mostly not advanced to clinical development. A major consideration for the translation of cell-based therapies is the ability to source cells ethically and frugally manufacture adequate quantities to support market demand. MSCs are the most intuitive option as they can be sourced from a range of tissues and theoretically can be expanded indefinitely. However, MSCs have been shown to lose potency upon passaging, which further hampers their implementation (76, 136). Also, MSCs are heterogenous and precise classification of MSCs has been controversial (137). It is possible that certain MSC subpopulations have differential effects on healing, and advanced sorting may be required. The donor characteristics such as age and health status also have a profound impact on MSC properties, making autologous therapies unlikely. In any case, allogenic MSCs have been shown to have low-immunogenicity and acceptable safety profiles (81). It is pertinent to establish inclusion criteria including passage number, surface antigens, donor age, and donor health status if cells are being gathered from a bank. Alternatively, iPSC technology presents an interesting avenue that might circumvent some of these obstacles. iPSCs can be used to generate MSCs and adult cells which might be suited for AAA treatment such as vascular progenitors, ECs, and VSMCs. Regardless, the feasibility of using any cell as a reliable clinical therapy is governed by the ability to develop a scalable process from harvesting and expansion, to cryopreservation and thawing, and ultimately therapeutic delivery.

**Table 1:** Summary of cell therapies by cell type.

Cell type	Animal model	Delivery method	Key findings	Reference
VSMCs or progenitors	Rat xenograft model	Endoluminal seeding	Preserved aorta diameter and elastinolysis (8 weeks); decreased MMP translation, except for MMP2 (1 and 2 weeks); increased TIMP/TIMP3 expressions (1 and 2 weeks)	Allaire et al. (57)
	Rat xenograft model	Catheter infusion	No increase in aorta diameter and preserved elastin content (8 weeks); increase in fibrous collagen transcripts (1 week)	Allaire et al. (58)
	Rat elastase model	Perfusion	No reports on cell therapy impact on AAA morphology; reduction in MMP9 activity and MMP2 and MMP9 gene expression (6 weeks)	Park et al. (59)
	Mouse elastase model	Periadventitial delivery (commercially-available collagen sponge, Vitene™)	Decrease in aorta diameter; increased medial contractile markers; reduced medial macrophages (4 weeks)	Mulorz et al. (60)
ECs	Rat xenograft model	Endovascular delivery	Reduced aorta diameter; reduction in inflammation and MMP-12 activity; establishment of a functional endothelium (4 weeks)	Franck et al. (74)
BM-MSCs	Mouse AngII model	Transplantation of BM-MSC sheets	Reduced aorta diameter; increase in elastin content; IGF-1 and TIMP-1 upregulation; downregulation of IL-6, MCP-1, and TNF-α (4 weeks)	Hashizume et al. (84)
	Rat xenograft model	Endovascular seeding	Reduced aorta diameter; decrease in MMP-9, increase in TIMP-1 (1 week); Increase in collagen and elastin content; reduction in monocyte and macrophage infiltration (4 weeks)	Schneider et al. (86)
	Mouse CaCl_2_/elastase model	Intravenous delivery	Cells were found to be localized in the aneurysm from the adventitial side; aneurysm attenuation; elastic fibers preservation; more detailed understanding of the mechanism of intravenous delivery (8 weeks)	Hosoyama et al. (87)
AD-MSCs	Rat CaCl_2_ model	Intravascular delivery	Improvement in elastin content; reduced MMP activity (4 weeks)	Tian et al. (90)
	Mouse elastase model	Periadventitial delivery (sponge connected to a catheter)	Reduced aneurysm progression; preserved elastic lamellae (2 weeks)	Blose et al. (91)
	Mouse elastase model	Intravenous delivery	Reduced inflammation and macrophage infiltration; inhibition of aneurysm progression (2 weeks)	Xie et al. (92)
	Rat CaCl_2_/elastase model	Periadventitial delivery (engineered scaffolds)	Prevented increase in aorta diameter; decrease in macrophage infiltration; increase in elastin and SMA (2 weeks)	Parvizi et al. (88)

Alternatively, cell-free treatment paradigms are more amenable to clinical translation (Table 2), but also have considerations that must be addressed. EVs provide many of the benefits of MSC therapies and can ideally be used an off-the shelf therapy yet are still accompanied by quality control issues such as purity and donor source. The feasibility of gene therapies to be manufactured on a global scale has already been demonstrated. Acellular biomaterials pose little concern when it comes to sourcing and clinical translation, however xeno-derived materials must be vetted for immunogenicity and batch variability. Overall, researchers must be mindful of the practical concerns and logistics associated with each treatment paradigm and develop methods that are suited for large scale clinical implementation.

**Table 2:** Summary of cell-free therapies.

Cell-free approach	Product	Key findings	Reference
EVs	MSCs- EVs	Inhibited NET-mediated ferroptosis in SMCs *in vivo*; inhibited AAA formation through NET formation prevention	Chen et al. (99)
	ADSC-Exos	Reduced elastin degradation via exosomal miR-17-5p	Hu et al. (100)
	MSC-EVs	Decreased inflammation and cytokine levels through miR-147-mediated downregulation of IL-17 and HMGB-1	Spinosa et al. (101)
	EVs-CKBP conjugate	Increased uptake of EVs; triggered pro-regenerative effect on cytokine activated SMCs *in vitro*	Sajeesh et al. (102)
Gene therapy	NPs with NF-κB siRNA	Reduced nitric oxide (NO) production, immune cell recruitment and cytokine release, and cell death; Reduction in AAA progression and rupture	Yan et al. (103)
	miR-199a-5p	Demonstrated that miR-199a-5p mediates Sirt1 expression, which induces VSMC senescence; identified several miRNA targets for downregulation in AAA patients	Tao et al. (105)
	LH1 AAV vector	Decreased AAA formation, progression, and rupture	Li et al. (25)
	BAF60c AVV vector	Reduced maximum aortic diameters and AAA development; no improvement in survival rates	Zhao et al. (107)
	lncRNA PVT1	Knocked down PVT1; attenuated aortic expansion, reduced VSMC apoptosis, matrix degradation, and inflammatory cytokine profile	Zhang et al. (108)
	lncRNA H19	Knocked down lncRNA H19; reduced AAA growth rate and VSMC apoptosis	Li et al. (109)
	lncRNA NEAT1	Knocked out NEAT1; reduced VSMC proliferative phenotype	Ahmed et al. (110)
	CRISPR	Developed CHO-PGEA/pCas9-sgFbn1 nanoparticles targeting Fibrillin-1; observed better uptake of NPs in aorta with AngII administration	Zhang et al. (112)
Targeted protein and drug delivery	TGF-β-hydrogel	Improved wall thickness and elastin integrity; increased transforming growth factor activated kinase (TAK1) and pSMAD2	Bai et al. (117)
	Doxycycline nanofibers	Reduced MMP2 and MMP9 activity; Reduced inflammatory cytokines; Improved elastin quality	Yamawaki-Ogata et al. (122)
	Batimastat anti-elastin NPs	Reduced MMP activity; improved histological features; prevented aortic dilation	Nosoudi et al. (123)
	PGG weeping endovascular balloon	Stabilized vascular matrix; attenuated expansion of AAA; reduced aortic diameter	Simionescu et al. (124)

### 5.3 Route of delivery

In addition to the numerous practical considerations in regenerative therapy design, route of delivery is perhaps the most critical for successful integration with existing clinical paradigms. Systemic infusion of cells or cell-free products provides the least invasive approach yet is limited by possible off target effects and poor target specificity. While direct intraluminal delivery affords easy integration into the clinical framework, any intraluminal approach must be engineered to prevent the payload from entering the circulation. Although ILT is often viewed as a barrier to intraluminal delivery, it plays a significant role in AAA biology and therefore might be a target to which therapies can be delivered to alter the course of aneurysm progression. Still, periadventitial approaches may be best suited for AAA pathology as the pathologic changes primarily occur in the wall, but these strategies must be tempered by the significant invasiveness of an aortic exposure for perivascular delivery. While a patient may be hypothetically spared the hemodynamic insult of an aortic cross clamp, as is required in open surgical repair, the impact of a regenerative therapy on hampering a future reoperation or surveillance imaging cannot be understated. Delivery strategies may also draw inspiration from the armamentarium of vascular surgeons in treating endoleaks after EVAR. Transarterial, translumbar, and transcaval approaches are all currently employed for delivery of coils or polymers with minimal morbidity. Adapting these techniques for intravascular or perivascular therapies could hold promise.

Regenerative therapies may also be directly paired with open surgical repair in designing vascular grafts. The goal of this strategy is not to regenerate the AAA but rather to improve the performance of grafts after surgical replacement. While the patency of aortoiliac synthetic grafts is acceptable, thrombotic complications and resistance to infection remain significant challenges, particularly in infected fields or mycotic aneurysm cases. Novel acellular vascular grafts that address these limitations have been employed for peripheral reconstruction and may offer similar advantages in aortic reconstruction for AAA (138, 139). There is also likely significant opportunity to pair biologically active therapies with endovascular therapies, as in the treatment of coronary or peripheral artery disease (140, 141). Though not yet employed in the clinical realm, modification of aortic stent-graft technologies presents one such strategy (142, 143). Pre-clinical efforts have largely focused on improving the performance of endografts (i.e., reducing endoleak) rather than regenerating the aneurysm itself. Given the existing clinical workflow and rise of an “endo first” approach, pairing regenerative and surgical therapies may hold significant promise. Modulating post-EVAR sac behavior is one intuitive goal as positive or negative remodeling already predicts late aortic rupture and long-term survival (134, 135). In the future, utilizing regenerative engineering principles to develop biologically active stent-grafts that incite AAA repair simultaneously present an exciting opportunity.

### 5.4 Mechanisms of action

Current regenerative approaches to treating AAA are premised on the therapeutic properties of exogenous cells. Unlike traditional pharmacological paradigms, the therapeutic effects of cells are not based on a single target or mechanism. Instead, cells offer a robust, multifactorial secretome that consists of growth factors, cytokines, ECM components, extracellular vesicles, and organelles, each with their own respective benefits to AAA healing. Current studies report reduced inflammation, oxidative stress, MMP activity and ECM fragmentation with increased cellularity, however it remains unclear how these effects are achieved. Carefully designed experiments must be conducted to determine the causal relationships between therapies and their respective outcomes. *In vitro* and *ex vivo* studies will help unravel some of these unknowns. Scientists should also take advantage of genetic knock-out, gene overexpression, and CRISPR-cas9 technologies. The advent of bulk and single-cell sequencing provides indispensable tools that can be employed to gain mechanistic insight and further deconvolute how regeneration is accomplished.

The most exciting healing mechanism is the potential for cell engraftment, replacing the cellular void left behind by degenerative changes. Currently, the fate of transplanted cells is unknown with conflicting reports of viability, survival, and differentiation. Prior studies have utilized clever experimental designs to distinguish whether tissue regeneration results from activation of endogenous cells, for example migration of neighboring cells into the anastomotic regions of xenografts, or from engraftment of transplanted cells. It is pertinent to conduct lineage tracing studies and incorporate live-cell *in vivo* tracking so that cell fate can be reliably predicted (144). The utilization of cells expressing reporter genes provides valuable insights into cell fate, albeit with challenges that warrant careful consideration. One potential limitation arises from false positives generated in the short term, attributed to macrophage phagocytosis. Additionally, genetic modifications intended for cell tracking may heighten immunogenicity, impacting cell potency and differentiation potential. Despite these challenges, genetically-labeled cells offer the advantage of combining with immunostaining techniques, enabling the identification of cell phenotype and spatial relationships. Advanced imaging techniques using contrast agents provide another avenue for cell tracking, offering compatibility with clinical imaging modalities and high sensitivity. However, they pose limitations for long-term tracking, as contrast agents persist post-cell death and are diluted with mitosis. The choice of imaging method should be a deliberate consideration based on the specific study objectives, with researchers exercising discretion in result interpretation, particularly in preclinical studies. In clinical studies, both genetic modification and contrast agent-based tracking encounter difficulties. Genetically modified cells face regulatory hurdles, while contrast agent-based tracking raises concerns about radiation exposure. Consequently, cell tracking is infrequently implemented in clinical trials. However, a robust understanding gained from preclinical studies can potentially mitigate the necessity for tracking cells in human subjects. Navigating these challenges requires a judicious approach, emphasizing the importance of choosing the most suitable method for a given study's objectives and exercising caution in result interpretation, particularly in the context of human applications.

### 5.5 Conclusion

In summary, AAA is a devastating progressive degenerative disease with confounding biological mechanisms that have made it challenging to develop new therapeutics using conventional strategies. Regenerative engineering is a burgeoning field well suited to AAA pathology as it aims to reverse tissue damage *in situ*. To date, current regenerative engineering approaches for AAA have been limited to cell therapies. Future studies are still critically necessary to further identify the mechanistic underpinnings of cell therapies. Alternatively, cell-free strategies are a promising new frontier with their own set of advantages and challenges. The substantial number of considerations in regenerative therapy design underscores the need for interdisciplinary collaboration. Determination of the therapeutic payload, route of delivery, mechanism of action, and desired positive outcome must each consider practical limitations for successful translation. Scientists, engineers, and vascular surgeons must each bring their individual expertise. We encourage continued research in this field and support imaginative regenerative engineering approaches that will ultimately result in translatable therapies that can be merged with standard practice.
